# Supplementary material for: The Food and Nutrition Environment at Secondary Schools in the Eastern Cape, South Africa as Reported by Learners
Source: Int J Environ Res Public Health. 2020 Jun 5;17(11):4038. doi: 10.3390/ijerph17114038 (PMC7312062; doi:10.3390/ijerph17114038)
Supplement: Supplementary file 1 [file ijerph-17-04038-s001.pdf]

Supplementary Table 1: NUTRITION KNOWLEDGE TEST
(Please tick the correct answer)

1. The key to healthy eating is to:

a)	Eat may different kinds of food	1
b).	Eat some food more than other food	2
c).	Eat certain kinds of food in moderation or in small amounts	3
d).	All the answers are correct	4

2.The following food items should not be eaten at all when one is trying to lose weight

a).	Bread and rice	1
b).	Meat and fish	2
c).	Margarine	3
d).	None of the answer are correct	4
e).	I do not know	5

3.From the list of groups of food below, which food items contain a lot of fibre?

a).	Oats, apples, beans	1
b).	Milk, yoghurt, cheese	2
c).	Beef, chicken, mutton	3
d).	Butter, margarine	4

4.	Eating different kinds of foods is healthier than eating only a few kinds of foods.	1. True	2. False	3. Don't know

5.	It is impossible to get all the vitamins and minerals you need from food, hence you need to take vitamins and mineral supplements.	1.True	2. False	3. Don't know

6.	To protect yourself from diseases, you should avoid eating many different kinds of food.	1. True	2. False	3. Don't know

7.	People who are overweight should not be physically active.	1. True	2. False	3. Don't know

8.	If you are eating a healthy diet, there is no need for you to be physically active.	1.True	2. False	3. Don't know

9.	If you want to lose weight, there is no need to be physically active, it is better to simply diet.	1. True	2. False	3. Don't know	

10. You should not have starches at most meals because

a).	They are not important for your health	1
b).	Even eating small amounts can cause weight gain	2
c).	They cause diseases	3
d).	None of the above answers are correct	4
e).	I do not know	5

11. From which group of food items should you eat the most every day?

a).	Bread, samp, rice, porridge	1
b).	Apples, bananas, spinach, carrots	2
c).	Milk, yoghurt, cheese	3
d).	Chicken, fish, beans, eggs	4
e).	Do not know	5

12. An example of a well balance diet consists of:

a).	Mostly meat with smaller amounts of starch, fruits, vegetables and dairy products	1
b).	Mostly vegetables and smaller amounts of meat and dairy products	2
c).	Mostly starches, vegetables and fruits with smaller amounts of meat and dairy products	3
d).	None of the above are correct	4

13. Which food has the most fibre?

a).	White rolls	1
b).	Brown bread	2
c).	White bread	3
d).	Whole wheat bread	4

14.	Starch foods should not be eaten when one is trying to lose weight.	True	False

15.	Eating bread is associated with weight gain.	True	False

16.	It is usually not necessary to wash vegetables before you cook them.	True	False

17. Which one of the foods listed would you eat the most if you were trying to increase fibre in your diet?

a).	Cakes and biscuits	1
b).	Apples and carrots	2
c).	Chips and pies	3
d).	Chicken and fresh fish	4

18. How many servings of fruits and vegetables should be eaten per day?

a).	One fruit and vegetable daily	1
b).	3-4 fruits and vegetables daily	2
c).	5 or more fruits and vegetables daily	3
d).	There is no need to eat fruits and vegetables daily	4

19. Which nutrients are found in large amounts in fruits and vegetables?

a).	Fibre and vitamin A	1
b).	Starches, fats and vitamin D	2
c).	Fats Iron and calcium	3
d).	None of the above	4

20. Which one of the foods listed would you eat the most if you were trying to increase fibre in your diet?

a).	Cakes and biscuits	1
b).	Apples and carrots	2
c).	Chips and pies	3
d).	Chicken and fresh fish	4

21.	Dry beans, peas and lentils should be eaten often	1. True	2. False	3. I don't Know

22.	Soya mince is as healthy as meat	1. True	2. False	3. I don't Know

23.	Dry beans, peas, lentils are a healthy choice to eat in place of meat	1. True	2. False	3. I don't Know

24. Cooked meat/fish/chicken sold on the street may not be safe to eat because

1).	It may have been undercooked	1	
2).	The cook may not have used fresh meat	2	
3).	It may have been kept for a long time before being cooked	3	
4).	All of the above are correct	4	

25. Which one of the food groups listed contains a lot of calcium?

1).	Chicken and eggs	1
2).	milk, yoghurt	2
3).	Pilchards	3
4).	2 and 3 are correct	4

26. How much milk or 'maas' should you have a day?

1).	None			1
2).	Half a cup			2
3).	One cup			3
4).	Two cups			4
27.	It is healthy to use only a little salt when cooking the	1. True	2.	3. Don't know

28. Which of the following food items has iodine been added?

1).	Bread	1
2).	Maize meal	2
3).	Table salt	3
4).	Powdered milk	4

28. Which of the following snacks is low in fat?

1).	Simba chips	1
2).	Popcorn	2
3).	Fried chips	3
4).	Nicknacks	4

30. Which breakfast menu contains little fat

1).	Whole wheat toast with thinly spread margarine	1
2).	Weet-bix with 2% fat milk	2
3).	Bacon and eggs	3
4).	One and two are correct	4

31.	It is healthy to use a lot of oil when cooking?	1. True	2. False	3. Don't know

32. How much water should you drink a day?

1).	You do not have to drink water every day	1
2).	One to three glasses a day	2
3).	4 to 6 glasses a day	3
4).	7 to 9 glasses a day	4
5).	I do not know	5

33.	You should add extra salt to your cooked food before you even eat it.	1. True	2. False	3. Don't know

34.	Any type of water is safe to drink.	1. True	2. False	3. Don't know

35.	Drinking a lot of wine, beer, cider can cause weight gain.	1.True	2. False	3. Don't know

36.	You should eat a lot of sugar to have enough energy.	1. True	2. False	3. Don't know
37.	What a pregnant woman eats during pregnancy has no effect on her health and the health of her unborn baby.	1. True	2. False	

38.	Sugar and foods containing sugar should be eaten in small amounts.	1. True	2. False	3. Don't know

39.	Sugar contains a lot of vitamins and minerals.	1. True	2. False	3. Don't know

40.	It is unhealthy to snack on food that contains a lot of sugar.	1. True	2. False	3. Don't know

Condensed from: Whati, L.H.; Senekal, M.; Steyn, N.P.; Nel, J.H.; Lombard, C.; Norris, S. Development of a reliable and valid nutrition questionnaire for urban South African adolescents. *Nutrition* **2005,** 21, 76-85.

Supplementary Table 2. The South African Food-Based Dietary Guidelines (FBDGs)

- Enjoy a variety of foods
- Be active
- Make starchy foods part of most meals
- Eat plenty of vegetables and fruits every day
- Eat dry beans, peas, lentils and soya regularly
- Have milk, *maas* (sour milk) or yoghurt every day
- Chicken, fish, lean meat, or eggs can be eaten daily
- Drink lots of safe, clean water
- Use fats sparingly**;** choose vegetable oils rather than hard fats
- Use sugar and food and drinks high in sugar sparingly
- Use salt and foods high in salt sparingly

Vorster, H.H.; Badham, J.B.; Venter, C.S. An introduction to the revised food- based dietary guidelines for South Africa. *S. Afr. J. Clin Nutr,* **2013,** 3, S5-S12.

4).	7 to 9 glasses a day	4
5).	I do not know	5

33.	You should add extra salt to your cooked food before you even eat it.	1. True	2. False	3. Don't know

34.	Any type of water is safe to drink.	1. True	2. False	3. Don't know	

3 5.	Drinking a lot of wine, beer, cider can cause weight gain.	1.True	2. False	3. Don't know	

.

36.	You should eat a lot of sugar to have enough energy.	1. True	2. False	3. Don't know	
37.	What a pregnant woman eats during pregnancy has no effect on her health and the health of her unborn baby.	1. True	2. False	3. Don't know	

38.	Sugar and foods containing sugar should be eaten in small Amounts.	1. True	2. False	3. Don't know	

39.	Sugar contains a lot of vitamins and minerals.	1. True	2. False	3. Don't know	

40.	It is unhealthy to snack on food that contains a lot of sugar.	1. True	2. False	3. Don't know	

c).	Pies, cakes puddings	3	
d).	None of the above	4	

21.	Dry beans, peas and lentils should be eaten often	1. True	2. False	3. I don't Know

22.	Soya mince is as healthy as meat	1. True	2. False	3. I don't Know

23.	Dry beans, peas, lentils are a healthy choice to eat in place of meat	1. True	2. False	3. I don't Know

27. Cooked meat/fish/chicken sold on the street may not be safe to eat because

1).	It may have been undercooked	1	
2).	The cook may not have used fresh meat	2	
3).	It may have been kept for a long time before being cooked	3	
4).	All of the above are correct	4	

28. Which one of the food groups listed contains a lot of calcium?

1).	Chicken and eggs	1
2).	milk, yoghurt	2
3).	Pilchards	3
4).	2 and 3 are correct	4

29. How much milk or 'maas' should you have a day?

1).	None	1	
2).	Half a cup	2	
3).	One cup	3	
4).	Two cups	4	

28. Which of the following food items has iodine been added?

1).	Bread	1	
2).	Maize meal	2	
3).	Table salt	3	
4).	Powdered milk	4	

29. Which of the following snacks is low in fat?

1).	Simba chips	1	
2).	Popcorn	2	
3).	Fried chips	3	
4).	Nicknacks	4	

30. Which of the following breakfast menu contains little fat?

1).	Whole wheat toast with thinly spread margarine	1
2).	Weet-bix with 2% fat milk	2
3).	Bacon and eggs	3
4).	One and two are correct	4

31.	It is healthy to use a lot of oil when cooking?	1. True	2. False	3. Don't know	

32. How much water should you drink a day?

1).	You do not have to drink water every day	1
2).	One to three glasses a day	2
3).	4 to 6 glasses a day	3
4).	7 to 9 glasses a day	4
5).	I do not know	5

33.	You should add extra salt to your cooked food before you even eat it.	1. True	2. False	3. Don't know

34.	Any type of water is safe to drink.	1. True	2. False	3. Don't know	

3 5.	Drinking a lot of wine, beer, cider can cause weight gain.	1.True	2. False	3. Don't know	

.

36.	You should eat a lot of sugar to have enough energy.	1. True	2. False	3. Don't know	
37.	What a pregnant woman eats during pregnancy has no effect on her health and the health of her unborn baby.	1. True	2. False	3. Don't know	

38.	Sugar and foods containing sugar should be eaten in small Amounts.	1. True	2. False	3. Don't know	

39.	Sugar contains a lot of vitamins and minerals.	1. True	2. False	3. Don't know	

40.	It is unhealthy to snack on food that contains a lot of sugar.	1. True	2. False	3. Don't know	

31. Which of the following food items has iodine been added?

1).	Bread	1	
2).	Maize meal	2	
3).	Table salt	3	
4).	Powdered milk	4	

32. Which of the following snacks is low in fat?

1).	Simba chips	1	
2).	Popcorn	2	
3).	Fried chips	3	
4).	Nicknacks	4	

33. Which of the following breakfast menu contains little fat?

1).	Whole wheat toast with thinly spread margarine	1
2).	Weet-bix with 2% fat milk	2
3).	Bacon and eggs	3
4).	One and two are correct	4

31.	It is healthy to use a lot of oil when cooking?	1. True	2. False	3. Don't know	

32. How much water should you drink a day?

1).	You do not have to drink water every day	1
2).	One to three glasses a day	2
3).	4 to 6 glasses a day	3

4).	7 to 9 glasses a day	4
5).	I do not know	5

33.	You should add extra salt to your cooked food before you even eat it.	1. True	2. False	3. Don't know

34.	Any type of water is safe to drink.	1. True	2. False	3. Don't know	

3 5.	Drinking a lot of wine, beer, cider can cause weight gain.	1.True	2. False	3. Don't know	

.

36.	You should eat a lot of sugar to have enough energy.	1. True	2. False	3. Don't know	
37.	What a pregnant woman eats during pregnancy has no effect on her health and the health of her unborn baby.	1. True	2. False	3. Don't know	

38.	Sugar and foods containing sugar should be eaten in small Amounts.	1. True	2. False	3. Don't know	

39.	Sugar contains a lot of vitamins and minerals.	1. True	2. False	3. Don't know	

40.	It is unhealthy to snack on food that contains a lot of sugar.	1. True	2. False	3. Don't know	
